# Strain Distribution in a Kennedy Class I Implant Assisted Removable Partial Denture under Various Loading Conditions

**DOI:** 10.1155/2013/351279

**Published:** 2013-04-30

**Authors:** Reza Shahmiri, John M. Aarts, Vincent Bennani, Raj Das, Michael V. Swain

**Affiliations:** ^1^Department of Oral Rehabilitation, Faculty of Dentistry, University of Otago, P.O. Box 647, Dunedin 9054, New Zealand; ^2^Department of Mechanical Engineering, University of Auckland, Auckland 1010, New Zealand

## Abstract

*Purpose*. This in vitro study investigates how unilateral and bilateral occlusal loads are transferred to an implant assisted removable partial denture (IARPD). *Materials and Methods*. A duplicate model of a Kennedy class I edentulous mandibular arch was made and then a conventional removable partial denture (RPD) fabricated. Two Straumann implants were placed in the second molar region, and the prosthesis was modified to accommodate implant retained ball attachments. Strain gages were incorporated into the fitting surface of both the framework and acrylic to measure microstrain (**μ**Strain). The IARPD was loaded to 120Ns unilaterally and bilaterally in three different loading positions. Statistical analysis was carried out using SPSS version 18.0 (SPSS, Inc., Chicago, IL, USA) with an alpha level of 0.05 to compare the maximum **μ**Strain values of the different loading conditions. *Results*. During unilateral and bilateral loading the maximum **μ**Strain was predominantly observed in a buccal direction. As the load was moved anteriorly the **μ**Strain increased in the mesial area. Unilateral loading resulted in a twisting of the structure and generated a strain mismatch between the metal and acrylic surfaces. *Conclusions*. Unilateral loading created lateral and vertical displacement of the IARPD. The curvature of the dental arch resulted in a twisting action which intensified as the unilateral load was moved anteriorly.

## 1. Introduction

A well-constructed removable partial denture (RPD) can be an adequate treatment option for the partially edentulous patient [1, 2]. The prosthesis is supported by the framework via teeth contact and by the distal extension base. The loading of the Kennedy class I is complicated by the mismatch of tissue resiliency and the abutment teeth which have different viscoelastic responses. The soft tissue under load has a displacement range of 350–500 *µ*m, whereas a sound tooth has a displacement of 20 *µ*m under the same load [3]. This mismatch of support will result in the transmission of torque forces to the abutment teeth via a rotational movement of the RPD [4]. In 1984 Watt and MacGregor [5] linked tooth mobility to the torque forces that are developed against the abutment teeth. In addition, the rotational movement of the RPD is directed towards the underlying soft tissue, and as a result the torque force in the soft tissue is then transmitted as a shearing force, which progressively causes resorption of residual ridges [6]. One of the recurrent problems associated with a bilateral distal extension RPD stems from the loading of the edentulous ridge [7]. While the above issues are detrimental to the patients tissues the RPD typically remains undamaged. Extensive research has been done on the design and materials used in RPDs. The RPD components and major connector have been fatigue tested and clinically assessed comprehensively. The most common failure of the RPD is predominantly due to fatigue of the clasp unit and incorrect casting or poor design of the framework [8–14].

The above issues associated with Kennedy class I RPDs have led to the use of posterior implants to resolve the biological issue. Placing two implant abutments distally in the mandible has been recommended to transform a Kennedy class I RPD into a tooth and implant assisted removable partial denture (IARPD) (or pseudo-Kennedy class III) [15, 16]. Implants in conjunction with a Kennedy class I RPD were used for the first time in the early 1970s [17], and since then clinical trials have indicated good implant survival rates [16–19]. Mijiritsky et al. [16] reported IARPD wearers had improved chewing ability and higher patient satisfaction. It is also well accepted that the use of implants to stabilize and support mandibular prostheses can increase maximum muscular function [15, 19–22]. However, several studies have reported complications, such as screw loosening, framework fracture, loosening of healing caps, fracture of framework, and/or acrylic denture bases [18, 23, 24].

Results from a multicenter randomized clinical trial of twenty-four patients fitted with an IARPD opposing a maxillary complete denture showed that after 12 months maintenance was required in 58.3% of the test group requiring intervention due to matrix activation/deactivation, loss of clasp retention, and fracturing of the IARPD acrylic [23]. Grossmann et al. [18] explained how important it is to consider the loading situation in the mandible because of the significant displacement of the denture base if it is not supported by the major connector. In a clinical report by Carpenter [25] it was reported that it would be best to construct a new RPD, indicating that this could avoid the major complication for IARPDs, which is the fracture of the acrylic around the implant attachment housing. Carpenter recommended the IARPD should be well reinforced, with metal around the location of the implant attachments. Kaufmann et al. [24], in an 8-year followup of implant supported removable appliances, which included IARPDs, found that technical complications were a regular occurrence. In the first year there was a very high rate of technical complications, mostly related to the anchorage system (matrices) of root copings and implants.

The cause of the complications can be linked to the additional forces placed on the prostheses and the altered support structure that needs to resist these forces. Verri et al. [26] used finite element analysis to measure the influence of the occlusal forces on IARPDs. They verified that there was no reduction in the tension forces on the abutment teeth, but there was a reduction in the support required from the posterior half of the residual ridge. 

Bilateral balanced occlusion is suggested as an option for distal extension IARPD, to evenly distribute forces across the prosthesis; however, such an occlusal design is not easily achievable especially when the antagonist arch is fully dentate [27]. There are currently no studies comparing the effects of bilateral versus unilateral loading on Kennedy class I IARPD. Ohkubo et al. [28] conducted a single blind randomized crossover study of 5 partially edentulous patients (Kennedy class I). The study showed that the masticatory force and contact areas were greater and more distally located following placement of an IARPD. Del'Arco Pignatta Cunha et al.'s [29] finite element analysis study of the influence of the location of the implants in association with an IARPD found that maximum stress was located around the implant in all situations.

This study investigates how unilateral and bilateral occlusal loads are transferred to the IARPD. The aim is to better understand the strain distribution in a Kennedy class I implant assisted removable partial denture under various loading conditions. The hypothesis is that the location of the occlusal load will not alter the way the strain is transferred to the IARPD.

## 2. Materials and Methods 

A mandibular clinical Kennedy class I situation was duplicated using two components, polyurethane and silicone. Polyurethane (Easycast) simulated the hard tissues (remaining teeth and bone). Easycast is a two-component rigid polyurethane compound with a Shore hardness scale of 65D. The Easycast was covered with a silicone layer (DeguDent, Deguform, Germany) to simulate the soft tissue. To standardize the experimentation the silicone covering the edentulous area was kept at a constant thickness of 2 millimetres. To achieve a uniform silicone layer the edentulous ridge areas of the mandibular model were covered with 2 mm of wax to simulate the maximum soft tissue thickness. A matrix was made with condensation silicone putty (Sil-Tech Ivoclar Vivadent AG, Schaan/Liechtenstein). After removing the wax, Deguform was injected into the matrix, and the uniform silicone layer was produced. The silicone material used has a Shore hardness A of 14 to 16, which is comparable to human soft tissue, which ranges from 16 to 21 [30].

The implants and the interface between the root and PDL were considered to be bonded; the movement of the teeth was not considered [31, 32]. The properties of the periodontal ligament (PDL) reported in the literature are highly variable. Ruse [33] highlights the flawed PDL modulus of elasticity values used in various FEA articles. The average hardness value for PDL-alveolar bone is 0.42 ± 0.1 GPa, alveolar bone is 0.56 ± 0.1 GPa, tubular dentin is 0.64 ± 0.1 GPa, cementum is 0.5 ± 0.1 GPa, and PDL-cementum is 0.5 ± 0.2 GPa [34]. Factors such as size and shape of the root, thickness, loading direction, and nonlinear properties make PDL difficult to consider [34, 35].

The model of the mandible was a rigid polyurethane compound with a Shore hardness D of 65. This study did not load the prothesis to failure so the influence of the PDL and cementum and bone hardness were considered to be minor. The volume ratio of trabecular bone to cortical bone depends on the type of bone and varies with age and gender. The mandible has been classified as having a flat bone structure and flat bone can have a ratio of 25 : 75 of cortical to trabecular bones. The overall stiffness of the bone was considered by taking the average, based on the relative volume of each bone type. It was calculated as follows:

(1) EAverage =[(volume  crotical×E-mod  crotical)    +(volume trabecular×E-mod trabecular)]  ×(volume crotical+volume trabecular)−1.

The elastic modulus 14.7 GPa for cortical bone and 0.49 GPa for trabecular bone was considered to be well represented by the rigid polyurethane compound used in this study [36].

A conventional cobalt-chrome molybdenum partial denture Kennedy class I RPD with a lingual bar, mesial occlusal rests, and I bar clasps on the first premolar abutments was fabricated (Wironit, BEGO, Bremer GoldschlägereiWilh, Germany). Vishay SR-4 general purpose strain gages (Vishay Electronic GmbH, Germany) were first placed on the fitting surface of the metal framework in a previously prepared slot to accommodate the strain gage thickness. In order to measure small changes in resistance, the strain gauges used had a Wheatstone bridge arrangement with a voltage excitation. The voltage recorded needed to be converted to a resistance change value and then ultimately to a strain value. To find the resistance changes from voltage excitation in the Wheatstone bridge, the gauge factor was calculated first. All values obtained from the strain gauges were placed in the equation below to calculate microstrain values. As the voltage excitation for the PowerLab system is 2.5 volts (*V*_in_ = 2.5) the equation was modified to

(2) $$\begin{array}{c} \varepsilon =\frac{2.5\times dV_{\text{out}}}{\text{GF}}. \end{array}$$

**Figure 1:**
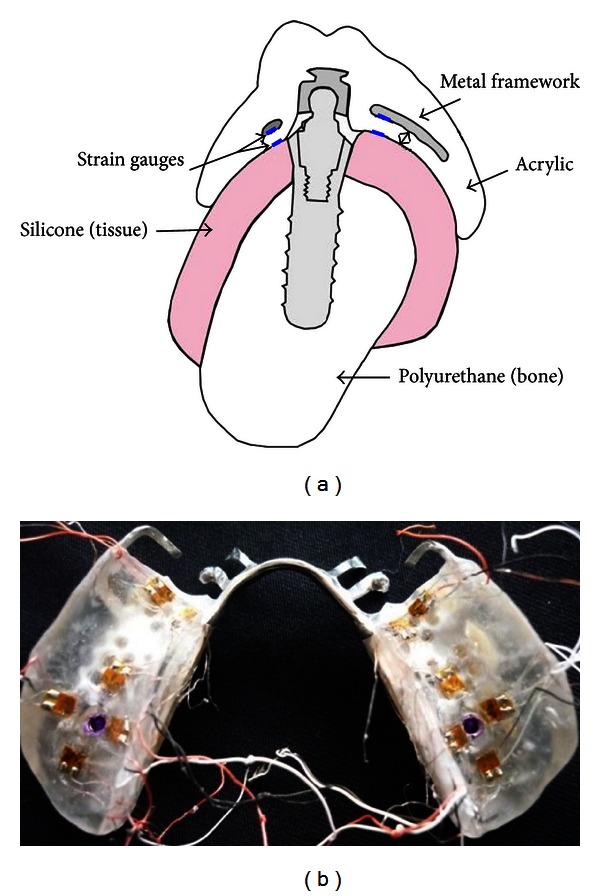
(a) Orientation of strain gauges on metal framework and acrylic surfaces. (b) Strain gauges placement on acrylic surface.

All strain gauges had a gauge factor of 2.07. Therefore, by placing the value of the output voltage in the strain equation, the value of strain could be calculated. The gauges type was 3057 CEA-06-015UW-120 (Vishay Electronic GmbH, Germany), with the temperature coefficient of resistance of +0.59% per degrees Celsius over a temperature range of +10 to +65 degrees Celsius (Vishay Technical Note Manual). The nominal resistance for the gauges was 120 Ω, and the gauges dimensions were 0.15 mm. Two strain gages were placed perpendicular to one another in the mesial area of the metal framework. A further four strain gages were placed on the metal framework, around each implant site. The teeth were placed, and clear acrylic was processed for the base. A further twelve strain gages were then placed on the acrylic surface with the same orientation as on the metal framework (Figures 1(a) and 1(b)).

The holes for the implants were prepared on the model using a milling unit. Drilling was performed parallel to the path of insertion of the partial denture. A 2.2 mm diameter pilot drill (number 044.211) (Straumann Group, SIX: STMN, Basel, Switzerland) was used initially, followed by a 2.8 mm diameter drill (number 044.216), and finally a 3.5 mm diameter drill (number 044.219). Standard regular neck (*Ø* 4.8 mm) ITI Straumann dental implants were placed into the model in the region of the first molar. 

 Easycast resin-based material was poured into the holes and the implants were screwed into place and left for 72 hours to cure. Ball attachments were then placed on the implants and screwed down. Then the retentive caps (Straumann titanium matrix, 048.450) were placed on the ball abutments, and the partial denture was relined with chemical-cured acrylic to secure the retentive caps in place.

**Figure 2:**
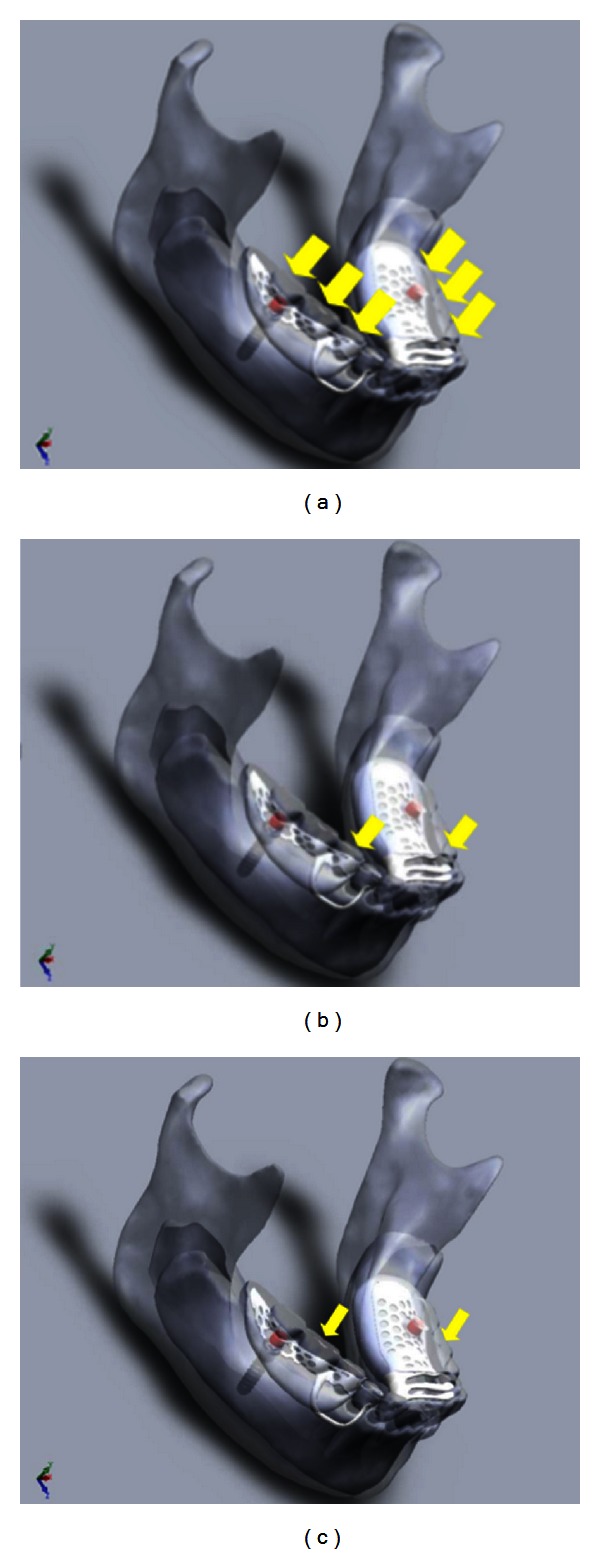
(a) Bilateral loading uniform; (b) bilateral loading premolar; (c) bilateral loading molar.

**Figure 3:**
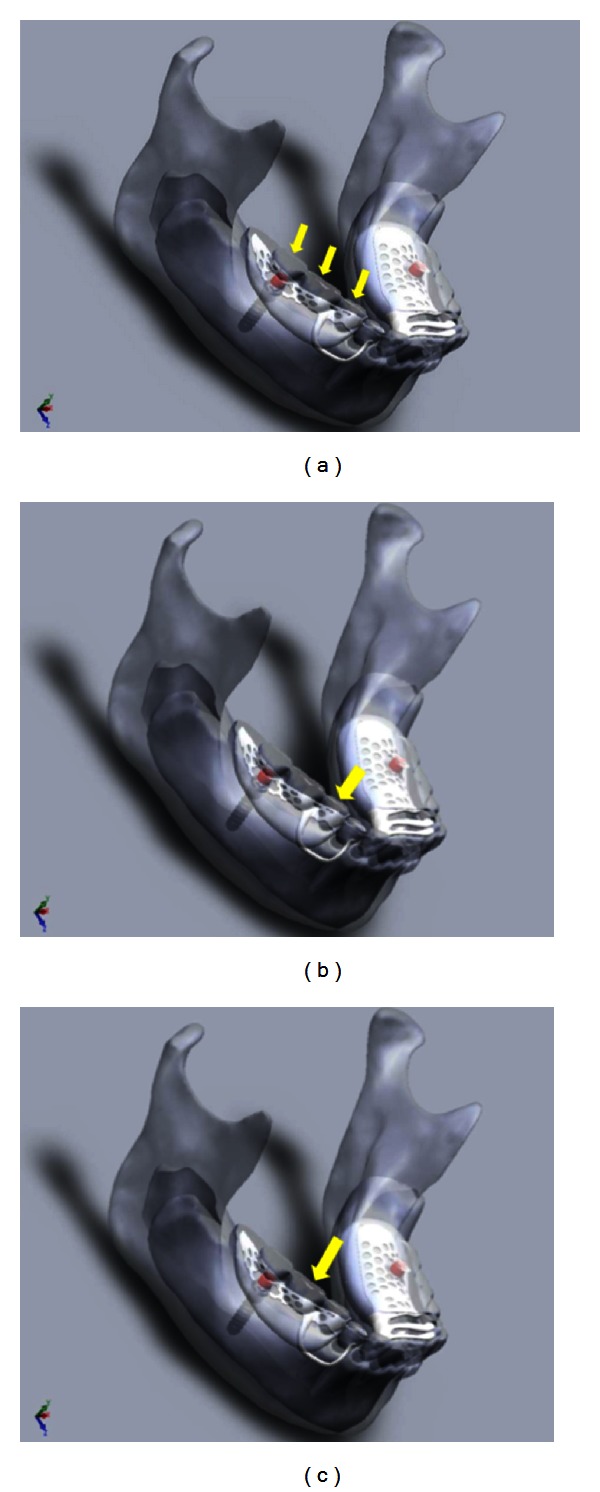
(a) Unilateral loading uniform; (b) unilateral loading premolar; (c) unilateral loading molar.

A load of 120 N was applied at a crosshead speed of 0.05 mm/sec using an Instron 3369 universal testing machine (Instron, Norwood, MA, USA). Two steel bars with different widths were used for the bilateral loading condition. A wider bar that covered all the denture teeth was used for the uniform loading, and a narrower bar was used to cover only the selected premolar and molar teeth. For the uniform unilateral condition a smaller bar was used. A thin silicone layer was placed between the bar and the teeth to distribute the forces more evenly. The silicone was placed on top of the teeth and a small amount of load was applied with the loading machine to ensure that even loading was achieved. The embedding of the bar into the silicone also minimized displacement of the steel bar during loading. A small V-shaped groove was created in the bar to locate the tip of the loading point accurately for the bilateral loading, whereas the uniform loading could be done by directly loading with a flat loading point fitted to the Instron (Figures 2(a), 2(b), 2(c), 3(a), 3(b), and 3(c)). The prosthesis was loaded, bilaterally and unilaterally, in the anterior premolar region, posterior molar region, and uniformly covering the premolar and molar regions. Each loading condition was repeated 10 times.

Loading was controlled using Instron Bluehill Lite Software. The data were detected as *µ*volts and recorded with Chart 5 software and PowerLab system (AD Instrument, Sydney, Australia). Data was presented as a graph and the highest points for all of the test cycles were manually recorded and converted to *µ*Strain. Statistical analysis was performed with SPSS version 18.0 (SPSS, Inc., Chicago, IL, USA) with an alpha level of 0.05 to compare the maximum *µ*Strain values of all three loading conditions. ANOVA was used to compare maximum mean *µ*Strain values identified in each loading condition.

## 3. Results

### 3.1. Microstrain on the Metal Surface during Bilateral Loading

Predominantly all the three loading situations produced high tensile *μ*Strain in the mesial area of the ridge in the buccal direction. Compressive forces were found in the anterior direction. Premolar loading produced lower *μ*Strain around the implants compared to the mesial area of the IARPD. Molar loading created moderate *μ*Strain around the implant and mesial area. Uniform loading developed a similar force distribution as per premolar loading, with a small decrease of *μ*Strain in the mesial area (Table 1).

**Figure 4:**
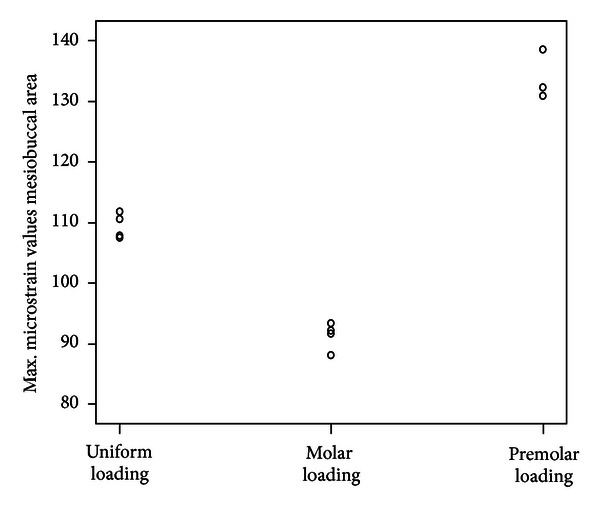
Maximum mean microstrain values of metal surface for all three bilateral loading conditions (left lateral mesiobuccal area).

Statistical analysis of the highest microstrain values of all three loading conditions showed that there were significant differences (*P* < 0.001) in the mesial area of the distal extension in the buccolingual direction. The maximum *μ*Strain values in the mesiobuccal area of the distal extension are shown in Figure 4 to illustrate consistency of data and provide a comparison of the acrylic areas. Among the three loading conditions, molar loading showed the lowest *μ*Strain values and had the most consistent maximum *μ*Strain. 

### 3.2. Microstrain on the Acrylic Surface during Bilateral Loading

Premolar loading produced high tensile *μ*Strain in the mesial area of the residual ridge in the buccal direction, while relatively high it was proportionally lower than the *µ*Strain around the implants. During molar loading higher tensile *μ*Strain was recorded in general, while compressive *µ*Strain was measured in the anterior direction in the mesial area of the IARPD. Premolar loading produced the highest tensile *μ*Strain around the implant area with moderate compressive *μ*Strain in the mesial area (Table 1).

Because both implant and mesial areas of the distal extensions showed high *µ*Strain values under different loading conditions, it was important to identify whether the differences were statistically significant. Statistical analysis was performed with SPSS 18.0 (SPSS, Inc., Chicago, IL, USA) to compare maximum *μ*Strain in all three loading conditions. ANOVA was used to compare maximum mean *µ*Strain for each loading condition. The comparison showed a highly significant increase in the mean *μ*Strain (*P* < 0.001) around the mesial area of the distal extension in the buccolingual direction as the loading point moved forward. In contrast, a significant decrease of the mean *µ*Strain (*P* < 0.001) was observed in the buccolingual direction around the implant area as the loading point moved forward.

**Figure 5:**
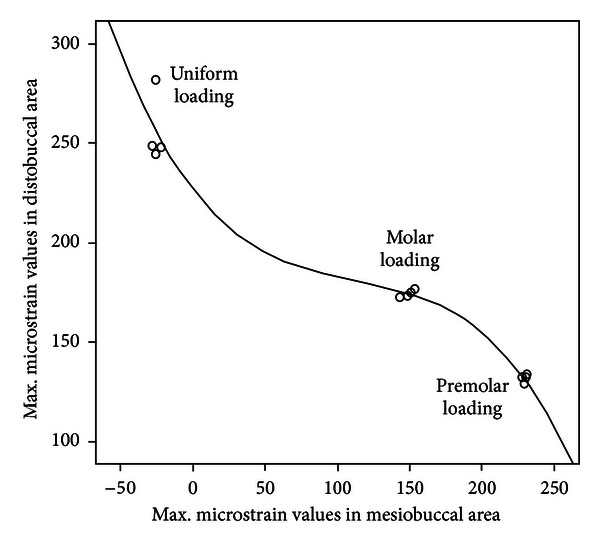
An inverse correlation between maximum microstrain of the mesiobuccal area next to the rest versus the distobuccal area next to the implant as the loading point moves from uniform to molar and then premolar.

A correlation analysis in Figure 5 shows the reverse correlation when moving the load forward with an increase in the maximum *μ*Strain (*R* = −0.985). During the bilateral loading, molar loading showed more even distribution of *µ*Strain around both the mesial and implant areas of the acrylic.

### 3.3. Comparison of Microstrain Behaviour of Metal Framework and Acrylic Base (Bilateral Loading Conditions)

**Figure 6:**
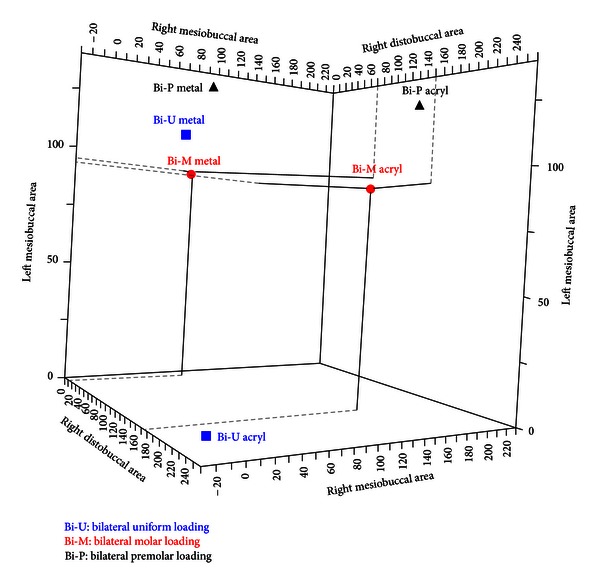
Comparison of microstrain values in both metal framework and acrylic base (bilateral loading conditions).

The maximum *µ*Strain values of the bilateral loading conditions for the framework and acrylic base were compared. This was done to identify the most favourable loading condition. Unscrambler X V10.1 software (The Unscrambler X, Camo, Norway) was used to draw a 3D scatter plot graph. Molar loading resulted in lower *μ*Strain and the best matching of the *µ*Strain between the framework and acrylic (Figure 6).

### 3.4. Microstrain on the Metal Surface during Unilateral Loading

Premolar loading produced tensile *µ*Strain in the anterior direction in the mesial of the metal framework, while compressive *µ*Strain was also evident in the buccal direction in that same area. Molar loading produced similar *µ*Strain patterns, but of a slightly lower magnitude. Uniform loading also produced similar *µ*Strain patterns as the premolar and molar loading, but produced the lowest *µ*Strain, except for the buccal gage around the implant (Table 1).

Of the three loading conditions, the highest *µ*Strain values were recorded in the mesial area of the distal extension. There were significant differences (*P* < 0.001) between the maximum *µ*Strain of all three loading conditions, and as the loading point moved forward there was a significant increase in the compression *µ*Strain.

### 3.5. Microstrain on the Acrylic Surface during Unilateral Loading

Premolar loading generated compressive *µ*Strain in the mesial of the ridge with higher *µ*Strain in the anterior direction. The *µ*Strain around the implant was tensile with higher *µ*Strain in the buccal and lingual directions. Molar loading produced the highest tensile *µ*Strain in the mesial area of the ridge in the buccal direction, but in the anterior direction under the same loading condition there was compressive *µ*Strain present. The area around the implant developed a more evenly distributed strain pattern. Uniform loading generated a very similar tensile pattern of *µ*Strain around the implant. Again a similar pattern was seen in the mesial area, but of a lesser intensity (Table 1).

### 3.6. Comparison of Microstrain Behaviour of Metal Framework and Acrylic Base (Unilateral Loading Conditions)

**Figure 7:**
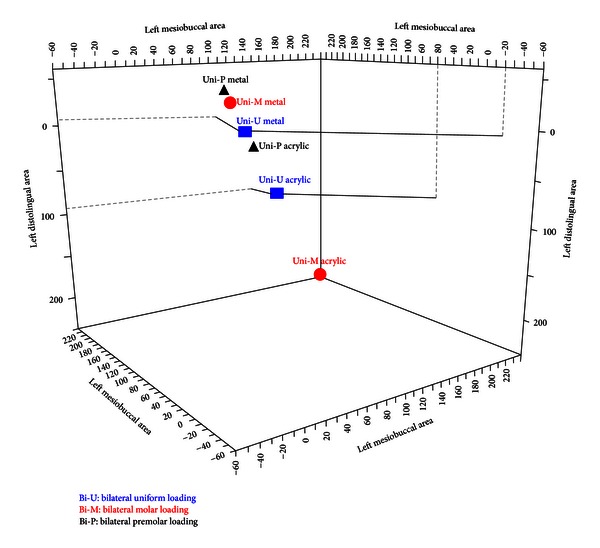
Comparison of microstrain values in both metal framework and acrylic base (unilateral loading conditions).

The uniform unilateral loading showed less *µ*Strain in both the framework and acrylic structures with more evenly distributed *µ*Strain than premolar and molar loading. However, there was a mismatch of strain type, the acrylic was in tension, and framework was in compression during all unilateral loading conditions (Figure 7).

## 4. Discussion

The focus of this in vitro study was to investigate how the IARPD distributed occlusal load, to better understand the way an IARPD transmits occlusal load. The incorporation of ball attachments into existing Kennedy class I RPD has led to subsequent increase maintenance of the IARPD. The area that appears to be most affected is predominantly around the implant attachment, resulting in attachment failure and/or acrylic fracture [18, 23, 24]. A Kennedy class I IARPD effectively alters a free end to a bounded prosthesis. This fundamentally changes the load dynamics and gives more stability and support to the patient, which in turn increases masticatory loading [15, 16, 20, 22, 37, 38]. 

The hypothesis that the location of the occlusal load will not alter the way the strain is transferred to the IARPD is rejected. Bilateral loading provides more stability and consequently generates more *με* on the occlusal rest arms. During unilateral loading of an IARPD the curvature of the dental arch created more displacement laterally as well as vertically. An off-axis lever is created, resulting in a twisting of the metal structure, and as the load was moved forward the effect of the twisting increased. The twisting also increased the tissue response against the acrylic and resulted in greater tension on the external surface of the acrylic. The metal structure is not in contact with the implant and twisting of the rigid lingual bar resulted in compressive *µ*Strain being developed on the metal surface. Since the implant attachment provides resistance to displacement most of the *με* was transferred to the areas surrounding the implant. 

This study has certain limitations: (1) the *μ*Strain patterns developed in IARPDs are complex, and the strain gauges measure the surface *µ*Strain at certain points and in pre-determined directions; (2) the implant osseointegration and the physiological mobility of the abutment teeth were not considered; (3) this study only looks at one IARPD design and does not consider the numerous designs that could be adopted. This study is only able to provide insight into the strain developed within this particular model; (4) controlled loading conditions were investigated, namely, unilateral and bilateral; unilateral loading imitated a fully dentate maxillary and bilateral loading simulated a patient with a complete maxilla denture. 

The physiological mobility of abutment teeth is unclear and potentially problematic to reproduce having one consistent material simulating the abutment and PDL produces more reliable results. In a finite element study by Ichim et al. [39] it was found that the strains in the mid-corpus and lower border of the mandible remain essentially the same regardless of the changes in the elastic modulus of the PDL. Each loading situation was tested 10 times on the IARPD; one test model was developed to limit the variables that could occur when using multiple models. The 120 N load selected represents a load relative to a standard bite force for a patient with an RPD [22] and was a load that the polyurethane model could repeatedly withstand without deforming. 

Loading the premolar and molar uniformly resulted in strain development throughout the IARPD, rather than local strain. As the loading point is moved forward, the strain is more evenly distributed between the two supporting structures. This strain pattern is typical of a beam structure, with forces inwardly directed relative to the implant and occlusal rest. The IARPD acts like a typical beam due to the implant and occlusal rest restraining it from vertical movement. Therefore, moving the load closer to a supporting structure should reduce the amount of deflection in the beam. However, the beam deflection behavior was not always present; this was due to additional resistance from the soft tissue on one side of the model. Increases in tissue displacement resulted in greater flexural resistance, and consequently, higher *µ*Strain developed on the acrylic surface. 

**Figure 8:**
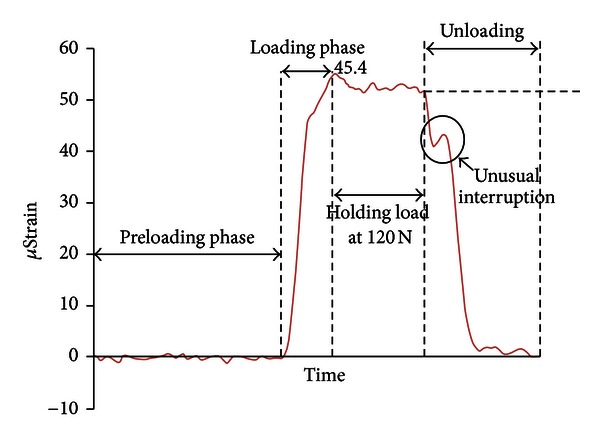
Microstrain-time plot of gauge 1 illustrating preloading, loading, and unloading microstrain behaviour during a bilateral uniform loading cycle.

There were significant differences (*P* < 0.001) between the maximum *µ*Strain values for the three loading conditions. The microstrain-time plot for the distobuccal gauge shown in Figure 8 provides further information about the microstrain behaviour during loading and unloading. 

During the loading phase, the microstrain-time curve changed its slope just prior to reaching a maximum value during the loading phase. The contacting of the supporting tissue during the loading phase limited the flexure of the denture and thereby reduced the gradient of *μ*Strain. A possible explanation is that as the load continued to increase, the prosthesis was in contact with the silicone, and further increase in load deflected both the acrylic and also the underlying silicone resulting in a lower rate of *μ*Strain increase. 

During the unloading phase there was an unusual interruption of microstrain identified in the plot graph. A gradual decline of microstrain values was observed during the initial unloading stage due to the recovery of the structure as the load was released. However, an interruption was noted during the unloading stage. This is consistent with an apparent partial reload during the unloading process. As indicated above, the acrylic made contact with tissue, so during unloading, initially the acrylic will recover, and the adhesion of the tissue will add additional force (the slight rise) before it releases.

**Figure 9:**
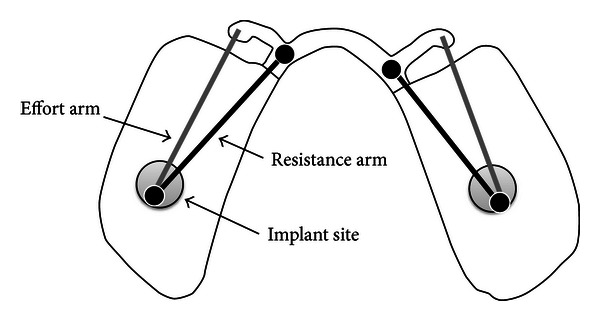
Lever created by the effort arm in relation to the resistance arm, demonstrating the increase in the lever as it extends anteriorly.

The loading of the structure was also influenced by the location of the load and the supporting structures, which creates a lever. The implant and rest (resistance arm) are not in alignment with the effort arm (Figure 9). Misalignment between the resistance arm and effort arm could possibly cause lateral displacement. Bilateral loading minimized lateral displacement through the rest arms, but moving the loading point forward increases the length of the effort arm and as a result generates greater mechanical advantage. This resulted in more displacement and a larger tissue response and consequently a greater *µ*Strain on the acrylic surface.

The *µ*Strain on the metal around the implant was not influenced by moving the loading area forward. This is understandable as the metal structure did not have direct contact with the implant, but it did have support via the occlusal rests placed on the abutment teeth. Hence, the occlusal rest arms support the metal structure. 

During bending the tensile forces are generated on the bottom surface of the structure and the top acrylic is put into compression. As acrylic is weak in tension and has a low elastic modulus, it is preferable that most of the flexural tensile forces are carried by the metal structure. During loading, the metal framework helps reduce the flexure of the structure and lowers the tensile *με* developed on the inner surface of the acrylic. Therefore, the rigidity of the metal structure in the neutral axis is important when limiting deflection. 

**Figure 10:**
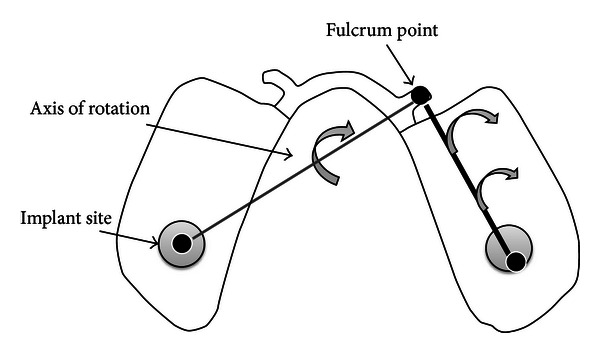
Twisting of the prosthesis around the axis of rotation during unilateral loading of right side.

In general, during unilateral loading the metal surface was subject to compressive *µ*Strain, and the acrylic was in tension. The implant attachment provides retention and additional resistance to displacement during unilateral loading resulting in the *μ*Strain being transferred to the areas surrounding the implant. There was a significant *μ*Strain increase in the buccal of the mesial ridge area, from 59.1 *μ*Strain to 234 *μ*Strain as the loading point was changed from uniform to the molar area. The *με* then changes from a tensile to a compressive strain when the load point was moved to the premolar area. In contrast to bilateral loading, which gives the prosthesis more vertical stability, unilateral loading can result in simultaneous vertical and lateral displacement. A possible explanation for the strain pattern seen during unilateral loading is the curvature of the dental arch. The occlusal rest arm on the same side as the load will no longer act as a vertical support, but rather act as a fulcrum point during displacement and cause twisting of the structure. Since the implant provides retention, it could be expected that most of the *μ*Strain will be formed around the implant during the twisting effect. However, the metal structure is not in contact with the implant and twisting of the very rigid lingual bar results in a compressive *μ*Strain being developed on the metal surface. This twisting will increase the tissue response against the acrylic and result in much greater tensile force on the external surface of the acrylic. Therefore, as the load moves forward, the effect of twisting becomes greater and results in greater compressive *µ*Strain occurring around the mesial area of the ridge (Figure 10).

This study reinforces the results found in Ohkubo et al. [20] which showed in a clinical study that there was an increase in distal loading after the placement of an IARPD. Del'Arco Pignatta Cunha et al.'s [29] finite elemental analysis study also identified that maximum stress was located around the implant area in IARPDs. It is clear that an IARPD creates a dynamic strain situation. This study illustrates that destructive strain patterns are present that could lead to the fracture of the IARPD acrylic.

## 5. Conclusions

- Unilateral loading created lateral and vertical displacement of the IARPD, which can result in strains and associated stresses on the major and minor connectors.
- Unilateral uniform loading of the IARPD generates a destructive strain correlation between the framework and acrylic base (acrylic in tension and framework in compression).
- The curvature of the dental arch created a fulcrum during unilateral loading which resulted in a twisting of the structure and as the load moved forward the effect of the twisting increased.
- Bilateral loading minimized lateral displacement, but when the load is moved forward the effect of the effort arm generated more strain on the occlusal rests.

## Figures and Tables

**Table 1:** Mean microstrain on metal and acrylic surfaces under different loading conditions.

			Mesial of the ridge				Around implant							
	Orientation of gage		Buccal direction		Anterior direction		Buccal direction		Lingual direction		Anterior direction		Posterior direction	
			Left side	Right side	Left side	Right side	Left ridge	Right ridge	Left ridge	Right ridge	Left ridge	Right ridge	Left ridge	Right ridge
Bilateral loading	Premolar *n* = 10	Metal	92.5 (2.3)	133.5 (3.4)	11.4 (1.2)	−33.2 (2.9)	4.7 (0.3)	2.8 (1.9)	−76.6 (1.9)	16.1 (2.1)	0 (1.2)	−23.6 (2.1)	1.0 (0.9)	−2.7 (1.8)
		Acrylic	228.7 (1.3)	127.9 (1.0)	6.5 (1.9)	−14.7 (2.2)	132.2 (2.0)	28.6 (1.0)	51.2 (1.2)	49.8 (3.7)	14.3 (1.6)	20.9 (1.6)	46.1 (3.8)	9.0 (2.3)
	Molar *n* = 10	Metal	70.3 (2.8)	91.3 (2.0)	6.6 (0.6)	−29.1 (1.7)	14.8 (0.4)	14.5 (2.2)	−79.1 (2.1)	27.2 (2.5)	4.4 (1.0)	26.0 (0.5)	0.2 (0.1)	2.1 (2.2)
		Acrylic	148.4 (4.5)	88.5 (0.7)	−2.0 (0.4)	−13.2 (2.5)	174.4 (2.7)	25.5 (1.8)	77.0 (2.3)	51.2 (1.0)	31.2 (1.4)	43.7 (1.1)	58.8 (4.5)	14.1 (0.4)
	Uniform *n* = 10	Metal	59.6 (2.9)	109.5 (2.0)	13.0 (1.8)	−46.1 (1.0)	3.9 (1.9)	9.7 (0.9)	−60.7 (2.8)	23.5 (1.0)	1.7 (1.2)	−5.4 (1.8)	0.4 (0.1)	−4.2 (1.9)
		Acrylic	−25.2 (2.1)	8.4 (4.2)	−22.6 (6.9)	−16.5 (10.1)	255.8 (15.2)	45.4 (11.7)	140.0 (2.1)	61.7 (4.7)	2.4 (4.0)	5.3 (2.7)	74.5 (4.7)	28.1 (4.0)

Unilateral loading	Premolar *n* = 10	Metal		−56.5 (0.8)		43.4 (4.9)		−2.1 (1.0)		−32.1 (1.8)		11.6 (0.7)		9.0 (0.5)
		Acrylic		−1.1 (0.4)		−46.1 (3.6)		46.5 (1.6)		87.7 (1.8)		0.6 (0.3)		13.6 (1.6)
	Molar *n* = 10	Metal		−47.1 (1.5)		32.4 (1.4)		−3.2 (0.6)		−21.5 (1.5)		7.0 (0.7)		6.3 (1.4)
		Acrylic		234.0 (2.6)		−34.6 (3.7)		37.6 (3.2)		97.6 (1.8)		76.5 (3.7)		21.0 (3.6)
	Uniform *n* = 10	Metal		−19.9 (2.9)		15.4 (2.3)		−6.2 (1.7)		−10.4 (2.1)		0.7 (1.4)		4.0 (1.9)
		Acrylic		59.1 (1.0)		−8.1 (3.2)		52.1 (1.4)		91.6 (1.4)		46.3 (3.1)		37.4 (1.6)

Negative *µ*Strain indicates compression.

Positive *µ*Strain indicates tension.

Standard deviation in brackets.
